# *Riemerella anatipestifer M949_0459* gene is responsible for the bacterial resistance to tigecycline

**DOI:** 10.18632/oncotarget.19633

**Published:** 2017-07-27

**Authors:** Tao Li, Min Shan, Jing He, Xiaolan Wang, Shaohui Wang, Mingxing Tian, Jingjing Qi, Tingrong Luo, Yonghong Shi, Chan Ding, Shengqing Yu

**Affiliations:** ^1^ Shanghai Veterinary Research Institute, Chinese Academy of Agricultural Sciences, Shanghai, China; ^2^ College of Animal Science and Technology, Guangxi University, Guangxi, China

**Keywords:** *Riemerella anatipestifer*, M949_0459 gene, MIC, tigecycline, resistance, Pathology Section

## Abstract

Based on its important role in last-line therapeutics against multidrug-resistant bacteria, tigecycline has been increasingly important in treating infections. However, mounting reports on tigecycline-resistant bacterial strains isolated from different sources are of concern, and molecular mechanisms regarding tigecycline resistance are poorly understood. *Riemerella anatipestifer* is a Gram-negative, non-motile, non-spore-forming, rod-shaped bacterium, which causes fibrinous pericarditis, perihepatitis, and meningitis in infected ducks. We previously constructed a random transposon mutant library using *Riemerella anatipestifer* strain CH3, in present study, we described that *Riemerella anatipestifer M949_0459* gene is responsible for the bacterial resistance to tigecycline. Using the minimum inhibitory concentration assay, a mutant strain showed significantly increased (about six-fold) tigecycline susceptibility. Subsequently, the knocked-down gene was identified as *M949_0459*, a putative flavin adenine dinucleotide-dependent oxidoreductase. To confirm the resistance function, *M949_0459* gene was overexpressed in *Escherichia coli* strain BL21, and the minimum inhibitory concentration analysis showed that the gene product conferred resistance to tigecycline. Additionally, expression of the *M949_0459* gene under treatment with tigecycline was measured with quantitative real-time PCR. Results showed that the mRNA expression of *M949_0459* gene was elevated under tigecycline treatment with dose range of 1-10 mg/L, and peaked at 4 mg/L. Moreover, two kinds of efflux pump inhibitors, carbonyl cyanide m-chlorophenyl hydrazone and phenylalanine arginyl *β*-naphthylamide were tested, which showed no function on tigecycline resistance in the strain CH3. Our results may provide insights into molecular mechanisms for chemotherapy in combating *Riemerella anatipestifer* infections.

## INTRODUCTION

Tigecycline, one member of the tetracyclines group called glycylcyclines, is a semisynthetic antibiotic with an expanded broad spectrum of potent activity against most of the clinically important bacterial pathogens, including many multidrug-resistant bacteria [1, 2]. As a tetracycline analog, a change in structure of an additional N-N dimethylglycylamido (DMG) group at the C_9_ position of the D ring of minocycline enhances the binding ability of tigecycline to its ribosomal target site and eludes common tetracycline resistance mechanisms [3]. Once, tigecycline was considered to be a last-chance antibiotic against multi-drug resistant bacteria. Since 2007, tigecycline has been suggested by the Food and Drug Administration for the treatment of complicated intra-abdominal infections, skin infections, and community-acquired pneumonia. Unfortunately, the widespread use of tigecycline has led to bacterial resistance. So far, the mechanisms of tigecycline resistance have mainly been investigated in *Klebsiella pneumonia*, *Escherichia coli*, and *Acinetobacter baumannii* [4-7].

*Riemerella anatipestifer* (*R. anatipestifer*)*,* a member of the *Flavobacteriaceae* family of the rRNA superfamily V [8], infects young ducks and geese, causing a severe form of Riemerellosis containing fibrinous pericarditis, perihepatitis, and meningitis. Riemerellosis is one of the most detrimental and lethal enteric diseases and is a major animal welfare and economic problem for the poultry industry. Because repeated infectious episodes are possible, eradication is difficult in duck flocks. Despite advances in novel vaccines, Riemerellosis has mainly been controlled by chemotherapy. Quinolones, tetracyclines, and cephalosporins are widely used for controlling *R. anatipestifer* infection in the avian breeding industry, and consequently have led to the emergence of antibiotic-resistant strains. Recently, some classes of drug resistance genes have been identified in *R. anatipestifer*, including aminoglycoside resistance genes (*ant*, *aac*, and *aph*), a chloramphenicol resistance gene (*cat*), and a chloramphenicol and florfenicol resistance gene (*floR*) [9-13].

Most studies of tigecycline focus on the bacteria found in humans, however, in many countries and areas, the variety of antibiotics used in the poultry industry is more than used in human medicine. In particular, in the avian breeding industry, the tigecycline analog tetracycline is commonly used and has resulted in increasing antibiotic drug resistance [14, 15]. To date, tigecycline-resistance mechanisms in bacteria in the *Flavobacteriaceae* family have not been studied. In our previous study, we constructed a random transposon insertion library using the *R. anatipestifer* strain CH3 [16]. In this work, using the minimum inhibitory concentration (MIC) assay of tigecycline, we obtained a mutant strain which showed a significantly increased (about six-fold) tigecycline susceptibility compared with the wild-type strain CH3.To clarify whether the increased tigecycline susceptibility is associated with the knocked-down gene, the mutant strain and the inactivated gene were characterized. Our results indicate that *R. anatipestifer M949_0459* gene is responsible for the bacterial resistance to tigecycline.

## RESULTS

### Identification of the mutant showing increased tigecycline susceptibility

**Figure 1:**
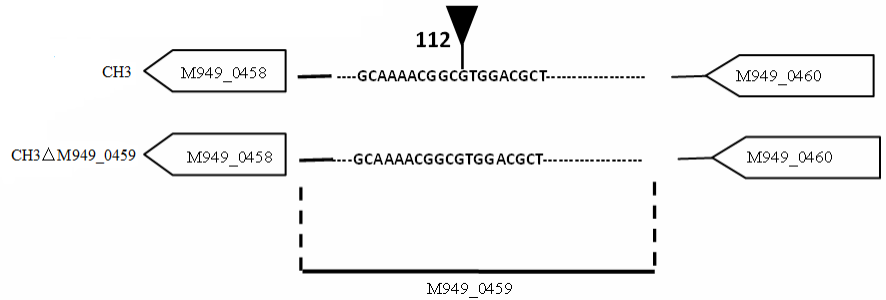
Schematic of the transposon insertion site The tip of infundibulum indicates the transposon insertion site (112 bp).

**Figure 2:**
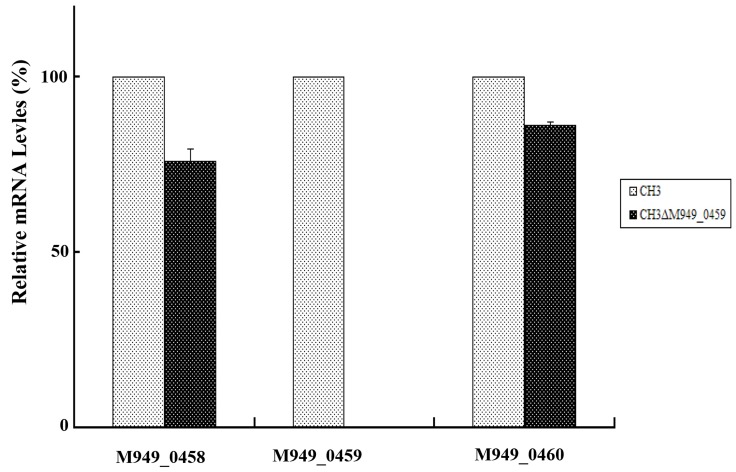
The *M949_0459* gene deletion did not affect expression of its flanking genes The expression of the *M949_0459* and its flanking genes in the mutant strain CH3ΔM949_0459 and wild-type strain CH3 was determined using quantitative real-time PCR analysis. The expression of *M949_0459* gene was inactivated in the mutant strain CH3ΔM949_0459, however, no significant difference was shown in expression of *M949_0458* and *M949_0460* genes between *R. anatipestifer* CH3 and CH3ΔM949_0459. Error bars represent standard deviations from three replicates.

In our previous work, about 2, 520 random transposon mutants were obtained [16], as mentioned above, using the tigecycline MIC assay, a mutant strain showed significantly increased (six-fold) tigecycline susceptibility (Table 2). With genome walking, the transposon-inserted gene was identified to be *M949_0459*, which encodes a putative flavin adenine dinucleotide (FAD)-dependent oxidoreductase of 386 amino acids, the mutant was named as CH3ΔM949_0459. The insertion of the transposon was located at nucleotide 112 of the gene (Figure 1). In addition, the effect of the *M949_0459* gene deletion on expression of the genes in the flanking regions was investigated using qRT-PCR. As shown in Figure 2, the *M949_0459* gene deletion inactivated the expression of the mutated gene, however, it did not affect the expression of both upstream gene *M949_0460*, which encodes the beta-lactamase class D protein, and the downstream gene *M949_0458*, which encodes a cation efflux protein, indicating transposon insertion in the *M949_0459* gene had no polar effect on its flanking gene expression. Moreover, a stability test of the mutant strain CH3ΔM949_0459 was performed based on the method of Fu and Tseng [20] with some modifications. The mutant strain CH3ΔM949_0459 was subcultured in a non-selective medium for more than 50 generations. Approximately 200 colonies were selected, and each colony was patched into TSA plates supplemented with or without erythromycin. No difference in growth was observed between the two types of plates.

**Table 1:** Strains, plasmids, and primers used in this study

Strains, plasmids or primers	Description	References
Strains		
CH3	*Riemerella anatipestifer* wild-type strain (serotype 1)	[49]
CH3ΔM949_0459	Mutant strain, derived from *Riemerella anatipestifer* CH3, in which the *M949_0459* gene was inactivated by Tn4351 insertion.	This study
plasmids		
Pet-28a(+)	Kan^r^, Bacterial expression vector with T7 lac promoter	[50]
Pet-28a(+)-M949_0459	Kan^r^, Bacterial expression vector with T7 lac promoter and the *M949_0459* gene insertion	This study
Primers		
RA 16S rRNA	5'-GAGCGGTAGAGTATCTTCGGATACT-3'	This study
RA 16S rRNA	5'-AATTCCTTTGAGTTTCAACCTTGCG-3'	This study
SP1	5'-CTCCCAGAAAATTTCCAAGACTCTCA-3'	This study
SP2	5'-TAAAGTGCTGACCCGTAAAACGAAC-3'	This study
SP3	5'-GTGGTAGCTATAGCATGGAGCTTGC-3'	This study
RTPCR-16SF	5'-CAACCATGCAGCACCTTGAAAA -3'	This study
RTPCR-16SR	5'-GACGAAAGCGTGGGGAGCGAAC-3'	This study
M949_0459 -F	5'-ACAAAGACCGAGATGCCAGG -3'	This study
M949_0459-R	5'-GTCAAACCGATTTTCGGGCT -3'	This study
M949_0458 -F	5'-GCTGTTGGCGGAACAGTAGT-3'	This study
M949_0458-R	5'-GTCAGGCATTTTCTCGACTCC-3'	This study
M949_0460 -F	5'-AGGAATGGTTTTCGATACTTTGACG-3'	This study
M949_0460 -R	5'-CCAGCCTGATTTCCCGTTCA-3'	This study

**Table 2:** Determination of bacterial susceptibility to antibiotics

Strains	Antibiotics							
	TGC MICs (mg/L)	AMP	NOR	CHL	CRO	ERY	GEN	KAN
CH3	12.0	S	I	R	S	S	R	R
CH3ΔM949_0459	2.0	S	I	R	S	R	R	R

Abbreviations: TGC, tigecycline; AMP, ampicillin; NOR, norfloxacin; CHL, chloromycetin; CRO, ceftriaxone; ERY, erythromycin; GEN, gentamicin; KAN, kanamycin. MIC, the minimum inhibitory concentration; S, susceptible; I, intermediate; R, resistant.

### Homologous gene and protein analyses.

**Figure 3:**
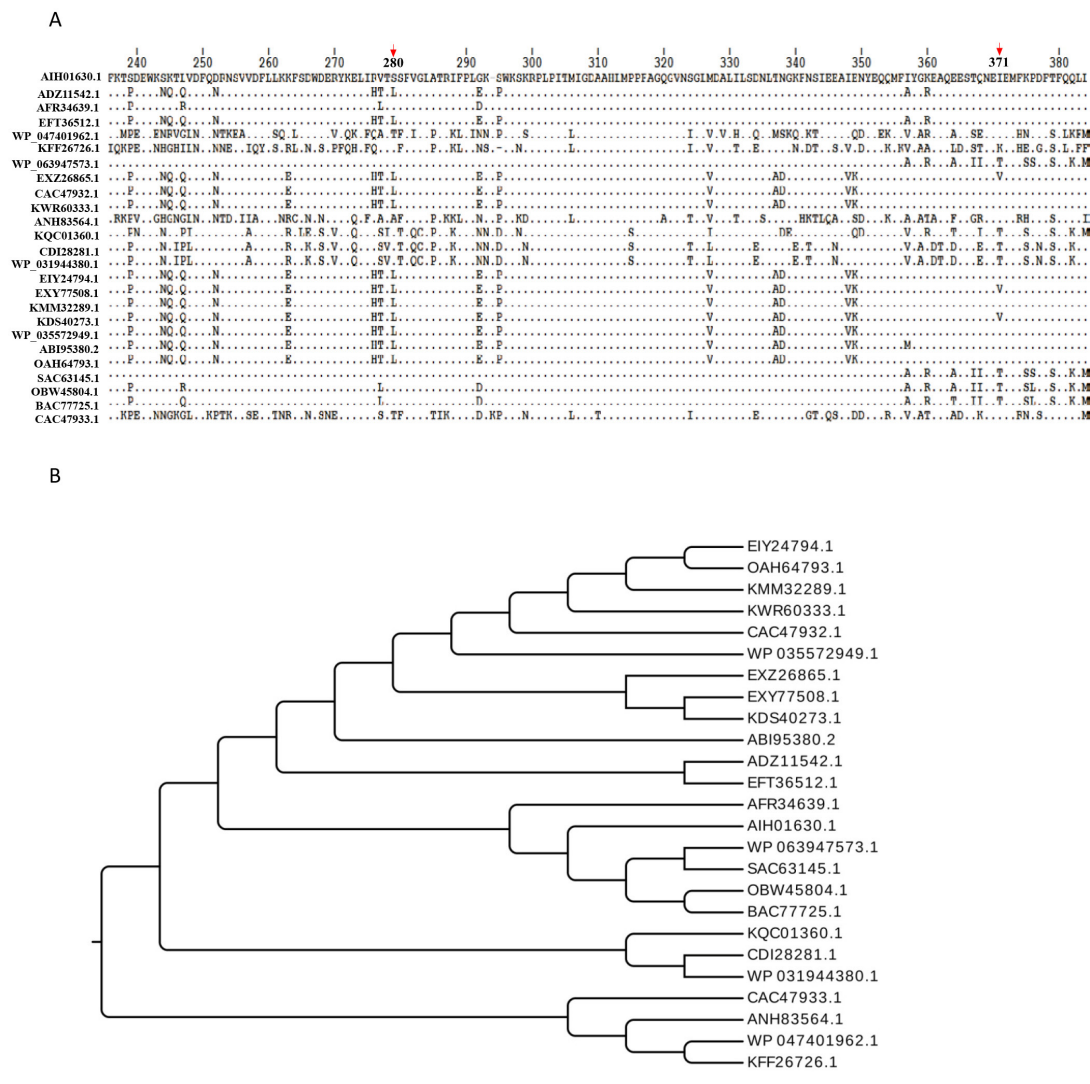
Bioinformatics analysis **A.** Multi-sequence alignment of the M949_0459 homologous proteins from different sources. The arrows indicate the locations of 280th and 371th amino acids. **B.** Evolutionary relation of the M949_0459 homologous proteins from different sources. Twenty five representatives were selected for the multi-sequence alignment and evolutionary relation analysis, including AIH01630.1 FAD-dependent oxidoreductase (*R. anatipestifer* CH3), ADZ11542.1 FAD-dependent oxidoreductase (*R. anatipestifer* RA-GD), AFR34639.1 hypothetical protein B739_0030 (*R. anatipestifer* RA-CH-1), EFT36512.1 monooxygenase, FAD-binding protein (*R. anatipestifer* RA-YM), WP_047401962.1 tetracycline resistance protein (*Chryseobacterium sp*. YR460), KFF26726.1 tetracycline resistance protein (*Chryseobacterium piperi*), WP_063947573.1 TetX family tetracycline inactivation enzyme (*Enterobacter cloacae*), EXZ26865.1 tetX2 protein (*Bacteroides fragilis* str. S36L11), CAC47932.1 TetX2 protein (*Bacteroides thetaiotaomicron*), KWR60333.1 kynurenine 3-monooxygenase (*Bacteroides ovatus*), ANH83564.1 tetracycline resistance protein (*Niabella ginsenosidivorans*), KQC01360.1 tetracycline resistance protein (*Pedobacter sp.* Hv1), CDI28281.1 FAD dependent oxidoreductase (*Acinetobacter pittii* 42F), WP_031944380.1 TetX family tetracycline inactivation enzyme (*Acinetobacter calcoaceticus*), EIY24794.1 hypothetical protein HMPREF1064_05159 (*Bacteroides dorei* CL02T12C06), EXY77508.1 tetX2 protein (*Bacteroides fragilis str.* 3988 T1), KMM32289.1 tetracycline resistance protein (*Parabacteroides goldsteinii*), KDS40273.1 tetX2 protein (*Bacteroides vulgatus str.* 3775 SRB19), WP_035572949.1TetX family tetracycline inactivation enzyme (*Elizabeth kingiaanophelis*), ABI95380.2 tetracycline resistance protein (*Sphingobacterium sp.* PM2-P1-29), OAH64793.1 tetracycline resistance protein (*Chryseobacterium sp.* J200), SAC63145.1 Kynurenine 3-monooxygenase (*Enterobacter cloacae*), OBW45804.1 Kynurenine 3-monooxygenase (*Chryseobacterium sp.* BGARF1), BAC77725.1 tetracycline inactivating enzyme (*Pseudomonas aeruginosa*), and CAC47933.1 TetX1 protein (*Bacteroides thetaiotaomicron*), respectively. The multi-sequence alignments were performed using CLUSTAL Omega.

**Figure 4:**
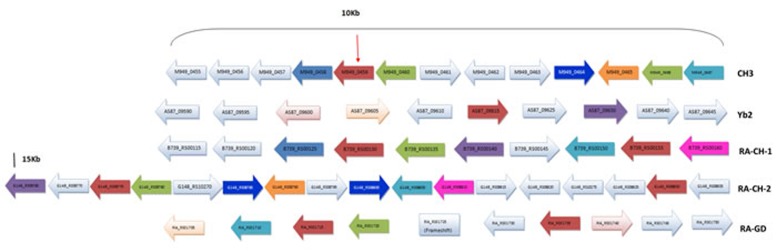
Gene environment around the *M949_0459* and its homology genes in *Riemerella* genomes The arrow indicates the *M949_0459* gene. The same color indicates the homology gene.

Currently, 28 complete *R. anatipestifer* genomes have been submitted to NCBI, BLAST analysis indicated that the *M949_0459* homologous sequence was observed in 6 of them (21%), suggesting that the distribution of the homologous sequence is limited in partial *Riemerella* strains. By BLASTP and FASTA algorithms, the M949_0459 presents 100% identity with G148_RS08775 and G148_RS08830 (RA-CH-2), 99% identity with B739_RS00155 (RA-CH-1), 95% identity with B739_RS00130 (RA-CH-1), 94% identity with RIA_RS01715 (RA-GD) and AS87_09615 (Yb2), and 93% identity with RIA_RS01735 (RA-GD). Multi-sequence alignment of the *M949_0459* homologous genes from different sources and evolutionary relation of the M949_0459 homologous proteins from different sources were summarized in Figure 3. Furthermore, comparative analyses of the *M949_0459* gene environment indicated that the upstream *M949_0460* homologous gene was observed in 4 of the 5 genomes, and the downstream *M949_0458* homologous gene was observed in 2 of the 5 genomes (Figure 4).

### Knock-down of the *M949_0459* gene decreased bacterial metabolic activity

**Figure 5:**
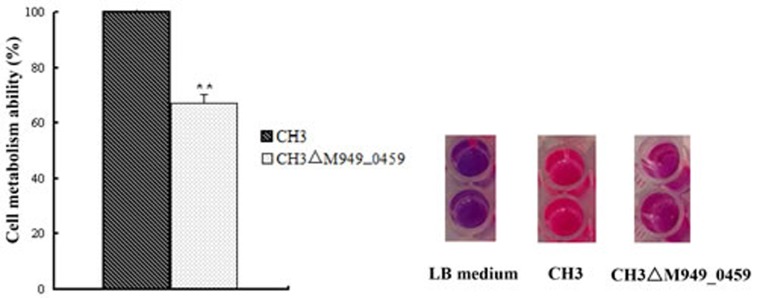
Knock-down of the *M949_0459* gene decreased bacterial metabolic activity The metabolic activity of the strains was measured using the alamarBlue reagent. **p < 0.01.

FAD-dependent oxidoreductase is a complex I NADH ubiquinone oxidoreductase that is crucial for transferring electrons into the respiratory chain in bacteria [21]. Mutations in FAD-dependent oxidoreductase may result in metabolic deficiencies. Based on the annotation of the M949_0459 as a putative FAD-dependent oxidoreductase in the GenBank, we tested the viability and metabolism of the bacteria using the alamarBlue assay. The results showed that the metabolic activity clearly decreased in the mutant strain CH3ΔM949_0459 compared with the wild-type strain CH3 (Figure 5).

### The *M949_0459* gene conferred tigecycline resistance to transformed *E. coli* BL21

**Figure 6:**
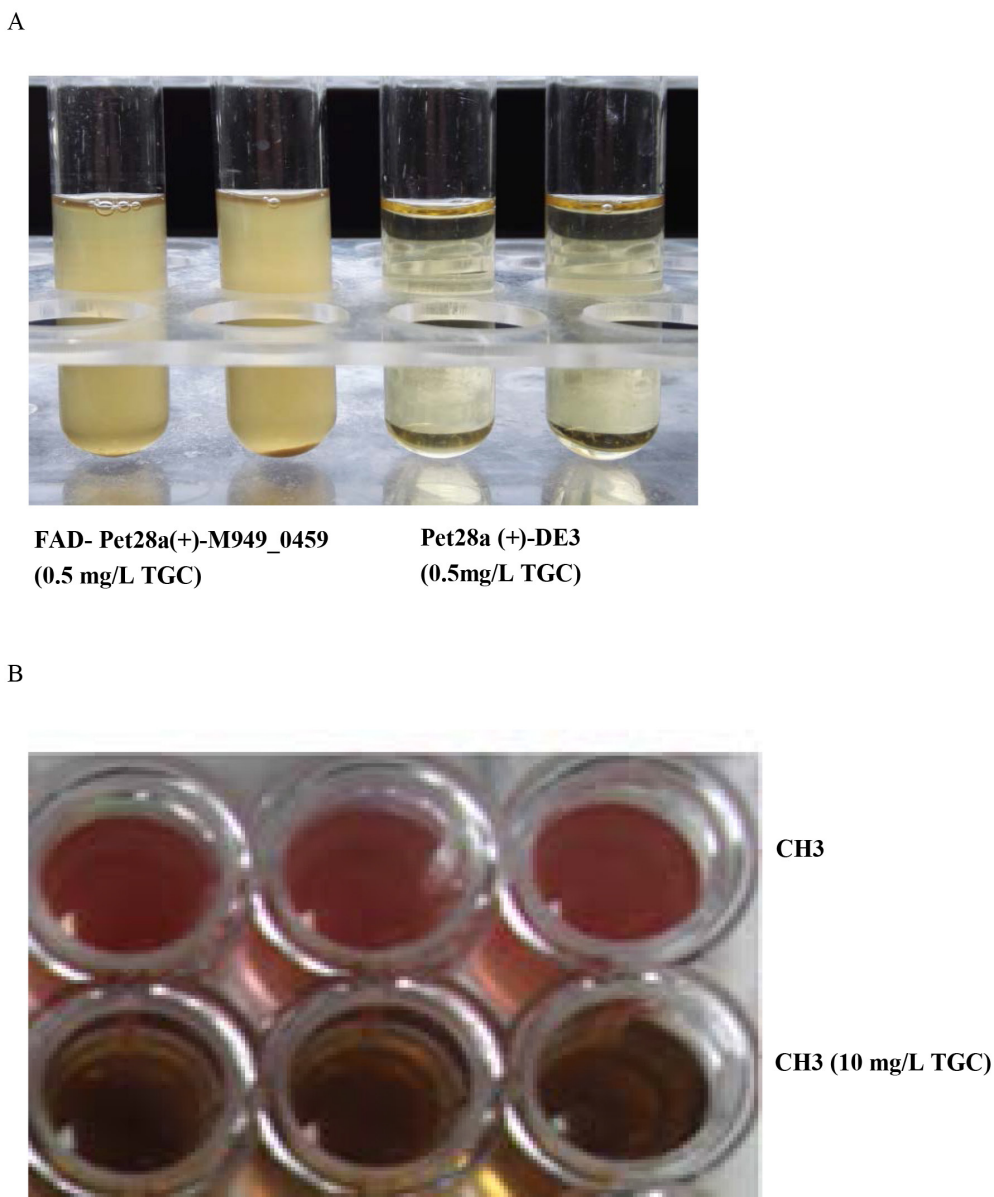
Determination of the tigecycline susceptibility of *E. coli* BL21 transformed with Pet-28a(+)-M949_0459 **A.** The transformants containing the *M949_0459* gene showed higher tigecycline resistance compared to controls. **B.** An oxygen-dependent color change was observed in the presence of high doses of tigecycline.

We measured the uptake of tigecycline by *E. coli* BL21 (DE3) which contained *M949_0459* on a Pet28a(+) background. The results showed that *M949_0459* conferred tigecycline resistance to *E. coli* BL21 (Table 3, Figure 6A) but did not affect susceptibility to other antibiotics including ampicillin, norfloxacin, ceftriaxone, gentamicin, kanamycin and imipenem. As mentioned above, sequence analysis of *M949_0459* revealed that this gene shared 91% similarity to *TetX* (*Bacteroides fragilis* str. S23L17). In 1988, Speer and Salyers first identified the *TetX* gene from a *Bacteroides fragilis* strain [22]*.* Further research discovered that TetX is a flavin-dependent monooxygenase that adds a hydroxyl group to the antibiotic to inactive tetracycline, and more importantly, with the presence of tetracycline, introduction of the *tetX* gene into *E. coli* induced a color change from orange to brown in the growth media [23]. Additionally, some evidence has shown that tigecycline and tetracycline act in a similar manner by binding to the same site on the ribosome [24, 25], our studies have indicated that an oxygen-dependent color change was observed in the presence of tigecycline (Figure 6B).

**Table 3:** Determination of the tigecycline susceptibility of *E. coli* BL21 transformed with Pet-28a (+)-M949_0459

Strains	Antibiotics						
	TGC MICs (mg/L)	AMP	NOR	CRO	GEN	KAN	IPM
Pet28a(+)-DE3	0.25	S	S	S	S	R	S
Pet-28a(+)-M949_0459	1.00	S	S	S	S	R	S

Abbreviations: TGC, tigecycline; AMP, ampicillin; NOR, norfloxacin; CRO, ceftriaxone; GEN, gentamicin; KAN, kanamycin; IPM, imipenem; MIC, the minimum inhibitory concentration; S, susceptible; R, resistant.

### Tigecycline treatment changed expression of the *M949_0459* gene in the bacteria at transcriptional level

**Figure 7:**
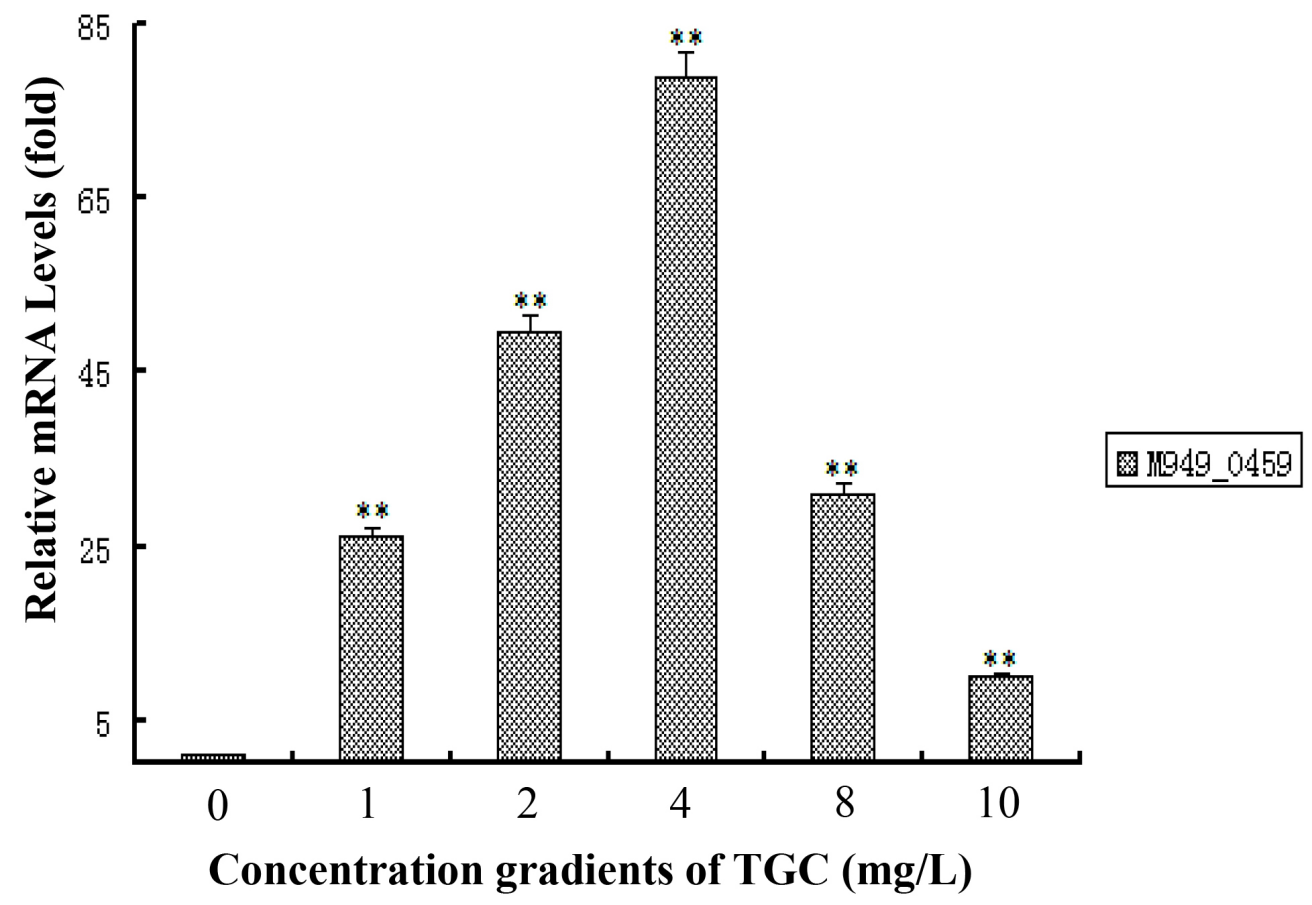
Tigecycline treatment changed the expression of *M949_0459* gene The expression of the *M949_0459* gene following treatment with different concentrations of tigecycline was measured by quantitative real-time PCR. Error bars represent standard deviations from three replicates. **p < 0.01.

By qRT-PCR, we quantitatively examined the expression of *M949_0459* following treatment with different doses of tigecycline. We observed a non-linear effect of the tigecycline dose on the *M949_0459* gene expression. The results showed that the mRNA expression of the *M949_0459* gene was elevated under tigecycline treatment with dose range of 1-10 mg/L, and peaked at 4 mg/L (Figure 7).

### The efflux pump inhibitors (EPIs) played no roles on the tigecycline susceptibility

In contrast to destruction or covalent modification of other antibiotics such as β-lactams and aminoglycosides, efflux systems have been identified as the primary mechanism of tigecycline resistance in *Klebsiella pneumonia*, *Proteus mirabilis*, and *E. coli* [26-28]. In this study, two kinds of EPIs, PA*β*N and CCCP, were used to evaluate the role of the efflux pumps in tigecycline resistance in *R. anatipestifer* strain CH3. As shown in Table 4, the tigecycline susceptibility of *R. anatipestifer* strain CH3 and CH3ΔM949_0459 showed no difference with or without the EPIs, indicating the efflux pump inhibitors (EPIs) play no roles on the tigecycline susceptibility.

**Table 4:** Effects of efflux pump inhibitors on tigecycline susceptibility

Strains	TGC MICs (mg/L)	
	Without EPIs	With inhibition of EPIs
CH3	12.0	12.0
CH3ΔM949_0459	2.0	2.0

Abbreviations: TGC, tigecycline; MIC, the minimum inhibitory concentration; EPIs, efflux pump inhibitors, including carbonyl cyanide m-chlorophenyl hydrazone (3 mg/L) and phenylalanine arginyl β-naphthylamide (40 mg/L).

## DISCUSSION

As a glycylcycline antimicrobial agent, tigecycline has been used for therapy in humans against a wide range of Gram-positive and Gram-negative anaerobic and aerobic bacteria and cell-wall free *mycoplasmas*, *chlamydiae*, and *mycobacterium* [29]. Although there have been no reports on the use of tigecycline against bacteria unique to a food animal, however, the first identified bacteria carrying special plasmids and antibiotic resistance genes were found in humans, which could make their way into food animal bacteria and vice versa. In many countries and areas, the total amount of antibiotics used in the poultry industry is more than the amount used in human medicine and is thus an important element in exposing bacteria to antibiotics [30-32]. In particular, in the avian breeding industry, the tigecycline analog tetracycline is commonly used and has resulted in increasing antibiotic drug resistance [33]. At present, increasing numbers of reports on tigecycline-resistant bacterial strains isolated from human sources are of concern, yet the molecular mechanisms regarding tigecycline resistance are poorly understood.

*R. anatipestifer* is ubiquitous in the environment, and has been isolated from ducks, turkeys, chickens, and other birds [9, 34]. In farmed ducks, *R. anatipestifer* infection leads to high mortality and consequently to great economic losses. Currently, a variety of approaches have been applied to control *R. anatipestifer* infections, but the most widely used strategy is chemotherapeutic agents in commercial poultry flocks, despite rising drug resistance. To date, several antibiotic resistance genes have been reported in *R. anatipestifer* [10, 11]. Using the tigecycline MIC assay, the *M949_0459* gene-deficient *R. anatipestifer* mutant strain CH3ΔM949_0459 had significantly increased tigecycline susceptibility compared with the wild-type strain CH3. Sequencing analysis revealed that the *M949_0459* gene encodes a putative FAD-dependent oxidoreductase. To confirm that the *M949_0459* is the resistance gene for tigecycline, *M949_0459* on a Pet28a(+) background was transformed into *E. coli* BL21 (DE3). As we had hypothesized, the expression of the recombinant protein conferred tigecycline resistance to *E. coli* BL21 (DE3) cells. Previous studies about the FAD-dependent oxidoreductase related to the resistance to the tigecycline analog tetracycline had demonstrated that the resistance mechanism of tetracycline was identified with detoxification of tetracycline, and resulted in a change in the color of the medium from yellow to gray-black [35]. In our current work, we showed a similar phenomenon in the *E. coli* BL21 (DE3) expressing *M949_0459*.

Although it is known that FAD-dependent oxidoreductase can alter tigecycline to 11a-hydroxytigecycline, resulting in a weakened ability to block protein translation [36], there have been no studies that demonstrate differences in FAD-dependent oxidoreductase protein expression induced by various doses of tigecycline. In our present work, the dose-response relationship between tigecycline and the mRNA level of *M949_0459* was measured. In the absence of tigecycline, there was detectable expression of *M949_0459*. At a 4 mg/L dose, there was a significant increase in expression, followed by a slight increase at 2 mg/L. However, when the dose of tigecycline increased from 4 mg/L to 10 mg/L, on the contrary, the *M949_0459* gene expression was gradually decreased, and finally was expressed at an extremely low level.

In addition to FAD-dependent oxidoreductase, resistance to tigecycline in the clinic also occurs through an efflux protein-mediated mechanism in bacteria. Efflux pumps can transport a specific substrate or a range of structurally dissimilar compounds. The latter efflux pumps are related to multidrug (antibiotic) resistance (MDR) [37, 38]. In bacteria, five families of efflux pump proteins have been described: the ATP-binding cassette superfamily, the major facilitator superfamily (MFS), the multidrug and toxic-compound extrusion (MATE) family, the small multidrug resistance (SMR) family, and the RND family [39]. Previous studies have shown that reduced tigecycline susceptibility mainly results from the overexpression of the RND pumps, AcrAB-TolC, and MATE pumps in different bacteria [40]. This led us to assess efflux pumps as an alternative tigecycline resistance mechanism in the current study. In our present work, two broad-spectrum EPIs, CCCP and PAβN, were used to identify the phenotype of active efflux systems. The results indicated that these two EPIs did not affect the susceptibility to tigecycline. Li *et al.* described the effect of CCCP and PAβN on the MIC of ten antibiotic agents against 66 strains of *R. anatipestifer* clinic isolates. They found that these two EPIs restored susceptibility of *R. anatipestifer* isolates to tetracycline [41]. Nevertheless, they did not examine the effect of these two EPIs on the susceptibility of *R. anatipestifer* isolates to tigecycline. It is possible that, different from tetracycline, tigecycline has a lower affinity for specific efflux pumps [42]. It is also possible that tigecycline cannot be expelled by MFS efflux pumps in either Gram-negative or Gram-positive bacteria [42]. Alternatively, it may be that different kinds of EPIs interfere with efflux pump activity through different modes of action [43]. Currently, no reports about MDR pumps in *R. anatipestifer* have been published, although genome analysis data has indicated that hypothetical MDR efflux pumps, such as those belonging to the MFS and SMR families, exist in *R. anatipestifer* [10, 44, 45]. Further research seeking specific resistance pumps playing important roles in extruding antimicrobial agents in *R. anatipestifer* is needed.

It is intriguing that the amino acid sequence deduced by *M949_0459* shares 91% sequence identity with the TetX2 protein (*B. fragilis* str. S23L17). Yang *et al.* first demonstrated that TetX2 belongs to a family of FAD-requiring monooxygenases that inactivate a broad selection of tetracycline antibiotics through reductive electron transfer or hydroxylation reactions [46]. Recently, Walkiewicz evaluated the potential of TetX tetracycline resistance proteins to acquire mutations causing tigecycline resistance. They found that among TetX mutants showing higher resistance levels to tigecycline, more than half contained the amino acid substitution as a single mutation or in combination with other changes at position 280. Furthermore, assessing the fitness of *E. coli* containing one copy of either wild-type TetX2 or variants of TetX2 amino acid substitution with a range of minocycline concentrations, the amino acid substitution T280A/T280S, and N371I/N371T were succeed in the bacterial drug adaptation experiment [47, 48]. In this study, a comparison of the sequences from M949_0459 and the other selected TetX sequences showed that the spontaneous amino acid substitution is present in M949_0459 at position 280 and 371 (Figure 3A).

In summary, we identified that *R. anatipestifer M949_0459* gene is responsible for the bacterial resistance to tigecycline, which may provide insights into molecular mechanisms for chemotherapy in combating *R. anatipestifer* infections.

## MATERIALS AND METHODS

### Bacterial strains, plasmids, and media

The bacterial strains and plasmids used in this study are listed in Table 1. *R. anatipestifer* wild-type strain CH3 and the mutant strain CH3ΔM949_0459 were used. The mutant strain CH3ΔM949_0459 was screened out from a random transposon mutant library that was previously constructed in our laboratory [16] using the tigecycline MIC assay. *R. anatipestifer* strains were grown in broth microdilutions according to the Clinical and Laboratory Standards Institute (CLSI) at 37°C. *E. coli* strains were grown at 37°C on Luria-Bertani (LB) plates or in LB broth. When necessary, the medium was supplemented with appropriate antibiotics at the following concentrations: ampicillin (100 mg/L), chloramphenicol (30 mg/L), erythromycin (0.5 mg/L), kanamycin (50 mg/L), and cefoxitin (5 mg/L).

### Antimicrobial susceptibility test

The MIC value of tigecycline was determined by serial dilution in 96-well culture plates, with these being performed according to the guide published by the CLSI in 2015. Other antimicrobial drug susceptibility tests were tested by the Kirby Bauer disc diffusion method according to CLSI guidelines in 2015.

### Identification of the mutant strain

The transposon insertion site in the mutant strain was determined with genome walking, which was performed with a genome walking kit (TaKaRa, Dalian, China) using four random primers (AP1, AP2, AP3, and AP4) provided in the kit and three specific primers (SP1, SP2, and SP3), according to the manufacturer’s instructions. The PCR-amplified gene was inserted into T-vector pMD19 (TaKaRa) for DNA sequencing (Shanghai HuaGene Biotech, Shanghai, China). The identified gene sequence was searched for homologous sequences on the BLAST server (http://blast.st-va.ncbi.nlm.nih.gov/Blast.cgi).

### Bioinformatics analysis

The COGnitor software (http://www.ncbi.nlm.nih.gov/COG/old/xognitor.html) was used to assess putative functions of the gene. The homologous protein sequences were searched using the BLASTX server (http://www.ncbi.nlm.nih.gov/BLASTX/) and aligned using CLUSTAL Omega [17].

### Quantitative real-time PCR analysis

Quantitative real-time PCR (qRT-PCR) was performed to measure the expression levels of the transposon-disrupted and flanking genes in wild-type strain CH3 and mutant strain, as well as the *M949_0459* gene following treatment with different doses of tigecycline at the transcriptional level. Total RNA of the samples was extracted with TRIzol reagent (Invitrogen, Carlsbad, CA, USA) according to the manufacturer’s instructions. To avoid DNA contamination, the extracted RNA was treated with RNase-free DNase I (40 U/mg RNA, TaKaRa) and purified using the RNeasy Mini Kit (Qiagen, Hilden, Germany) according to the manufacturer’s instructions. cDNA was synthesized using the GoTaq qPCR Master Mix Kit (Promega, Madison, WI, USA) following the manufacturer’s instructions after adjusting the samples to the same concentration. qRT-PCR was performed with the ABI7500 Real-Time PCR System (Applied Biosystems, Foster City, CA, USA). Gene-specific primers were designed using the primer3 online software v.0.4.0 (http://bioinfo.ut.ee/primer3-0.4.0/) and are described in Table 1. The expression of 16S rRNA was used as an internal control. The specificity of amplification was confirmed using melting curve and electrophoresis analysis. The data were analyzed with the normalized gene expression method (2-ΔΔCT) [18]. Each reaction was performed in triplicate, and the entire experiment was carried out in triplicate.

### alamarBlue assay

The metabolic activity of the strains was measured using the alamarBlue reagent (Thermo Fisher, Waltham, MA, USA), according to the manufacturer’s protocol. In brief, 20 µL of the alamarBlue reagent was directly added to 200 µL of the bacterial culture medium and blank medium (negative control); then, these were incubated for 1 h at 37°C while protected from direct light. Next, the absorbance of alamarBlue was monitored at 570 nm, using 600 nm as a reference wavelength (normalized to the 600nm value). The proliferation rate (r) was calculated according to the following formula: [117, 216×A_570_ (sample) - 80, 856×A_600_ (sample)] / [117, 216×A_570_ (control) - 80, 856×A_600_ (control)] × 100%.

### Construction and transformation of the Pet-28a(+)-M949_0459 plasmid

The DNA fragment carrying the *M949_0459* gene was amplified from the *R. anatipestifer* strain CH3 using the primers listed in Table 1. After amplification, the amplimer was cloned into the expression vector Pet-28a(+) to construct Pet-28a(+)-M949_0459 plasmid, and *E. coli* strain BL21 was subsequently used for transformation. Using Luria-Bertani agar (Difco, Detroit, MI, USA) containing kanamycin (50 mg/L), the transformants were selected and sequenced. The confirmed transformants were subjected to tigecycline susceptibility testing. *E. coli* BL21 with Pet-28a(+) vector without the DNA insertion was used as a control.

### Efflux pump inhibition assay

An efflux pump inhibition assay was performed as described previously [19]: broth microdilution testing of tigecycline was performed with and without phenylalanine arginine β-naphthylamide (PAβN) (40 mg/L) and carbonyl cyanide m-chlorophenyl hydrazone (CCCP) (3 mg/L).

### Statistical analysis

Statistical analysis was performed with Student’s *t* test. Mean values are shown in the figures. Statistical significance was established at *p* < 0.05.
